# Calponin 3 is associated with poor prognosis and regulates proliferation and metastasis in osteosarcoma

**DOI:** 10.18632/aging.103224

**Published:** 2020-07-14

**Authors:** Fei Dai, Fei Luo, Rui Zhou, Qiang Zhou, Jianzhong Xu, Zehua Zhang, Jun Xiao, Lei Song

**Affiliations:** 1 Department of Orthopaedics, First Affiliated Hospital, Army Medical University, Chongqing 400038, China; 2 Department of Orthopaedics, Third Affliated Hospital, Medical University of Chongqing, Chongqing 401120, China

**Keywords:** calponin 3, osteosarcoma, cell proliferation, metastasis, diagnostic and therapeutic target

## Abstract

Osteosarcoma is a malignant, life-threatening tumor that affects children and adolescents. In this study, we identified high levels of calponin 3 (CNN3) protein in osteosarcoma tissues and cell lines. The receiver operating characteristic curve analysis revealed that CNN3 has diagnostic value for patients with osteosarcoma. We also found that high CNN3 expression was associated with tumor size, tumor stage, and lymph node and distant metastases. Moreover, high levels of CNN3 mRNA were associated with a poor overall survival rate and a shorter disease-free survival period. CNN3 silencing inhibited cell proliferation, induced apoptosis and cell cycle arrest at the G1 stage, and inhibited cell migration and invasion *in vitro*. Furthermore, CNN3 silencing also inhibited subcutaneous tumor growth and lung metastasis *in vivo*. Western blotting revealed that silencing of CNN3 resulted in downregulated expression of MMP9, VEGF, and vimentin, and upregulation of E-cadherin. CNN3 silencing also resulted in downregulation of the ERK1/2 and p38 signaling pathways. In conclusion, high CNN3 expression was found to help in the diagnosis of osteosarcoma, and was found to be associated with poor prognosis in patients. Therefore, CNN3 may play an oncogenic role during the progression of osteosarcoma by activating the ERK1/2 and p38 pathways.

## INTRODUCTION

Osteosarcoma is a common childhood cancer [1, 2]. Currently, it is mainly treated using limb salvage, which is conservative in more than 90% of the patients [3]. The five-year relative survival rate for osteosarcoma can reach 69.8% or 65.5% for patients (at birth until 14 years of age or at 15 until 19 years of age) in the United States [4]. Notably, approximately 40–50% of the patients with osteosarcoma have pulmonary metastases, and most patients with osteosarcoma often have lung micrometastases before diagnosis. For such patients, chemotherapy, surgery, and/or other treatments are ineffective as the five-year relative survival rate for osteosarcoma patients with lung metastases is only 17–23% [5]. Therefore, the identification of diagnostic and therapeutic targets is necessary for the early detection and improved treatment of osteosarcoma.

Calponin 3 (CNN3) is a member of the CNN protein family, which contains tandem repeating sequence motifs [6]. CNN3 has a markedly acidic C-terminus, and its basic N-terminus is highly homologous to the N-terminus of CNN1, another member of the CNN protein family. Proteins belonging to this family are associated with the cytoskeleton but are not involved in contraction [7]. Apart from participating in the remodeling of actin stress fibers [8], members of the CNN protein family regulate carcinoma progression (e.g., in gastric and ovarian cancers) [9, 10]. However, their role in the progression of osteosarcoma has not been fully elucidated. Guo et al. reported that CNN3 expression levels were higher in human osteosarcoma cell lines than in SV40- immortalized normal osteoblastic cell lines [11]. Studies have recently shown that CNN3 functions as an oncogene in gastric, colon, and cervical cancers [12–14], and as a diagnostic marker of metastatic lymph nodes in colorectal cancer [15]. Consequently, CNN3 may serve as a new potential diagnostic tool and therapeutic target for the early detection and treatment of osteosarcoma.

In the present study, we aimed to investigate the role of CNN3 in regulating osteosarcoma progression. We determined the potential role of CNN3 as a biomarker for osteosarcoma diagnosis and prognosis and analyzed its function in regulating the proliferation and metastasis of osteosarcoma cell lines *in vitro* and *in vivo*. Additionally, we investigated the effects of CNN3 on the ERK1/2 and p38 signaling pathways in order to understand the mechanism underlying its activity.

## RESULTS

### High CNN3 expression and diagnostic performance in osteosarcoma tissues

**Figure 1:**
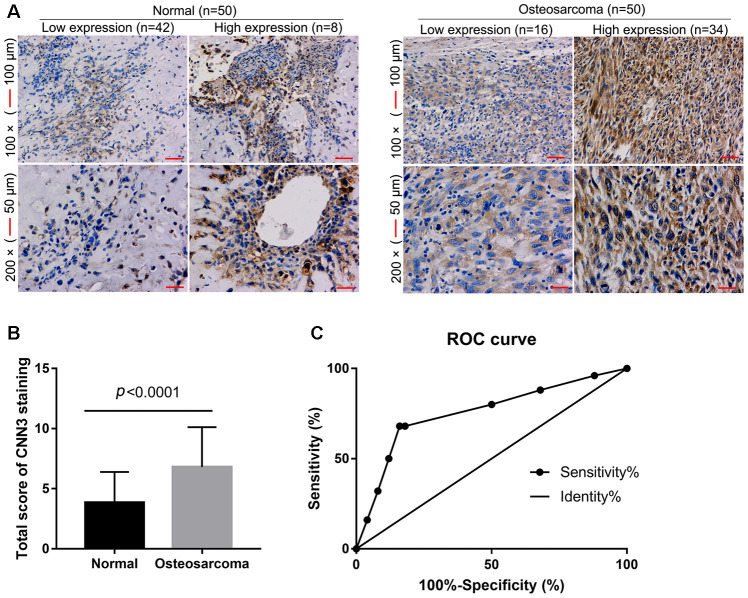
**CNN3 expression in osteosarcoma and normal specimens detected by immunohistochemistry.** (**A**) Representative images. (**B**) Statistical results of the CNN3 staining total score. *p*<0.0001, unpaired *t*-tests. (**C**) Receiver operating characteristic (ROC) curve analysis to assess the diagnostic value of CNN3 in osteosarcoma using the GraphPad Prism 7.0 version.

To assess the accuracy of CNN3 as a diagnostic biomarker for osteosarcoma, CNN3 expression levels in osteosarcoma and normal specimens (n=50 each) were detected by immunohistochemistry (IHC). IHC revealed that eight normal specimens have high CCN3 expression, and 34 osteosarcoma specimens have high CCN3 expression (*p*<0.0001, Figure 1A). The total score for CNN3 staining in osteosarcoma specimens was significantly higher than that in normal specimens (Figure 1B). These demonstrate that CNN3 is highly expressed in osteosarcoma tissues. Furthermore, the performance of CNN3 with respect to the diagnosis of osteosarcoma was analyzed using an ROC curve analysis; the area under the curve was 0.756. At the optimal expression cutoff value of 5.5, the sensitivity was 68% and the specificity was 84% (Figure 1C).

### Correlation of CNN3 protein levels with clinicopathological features and prognosis

**Figure 2:**
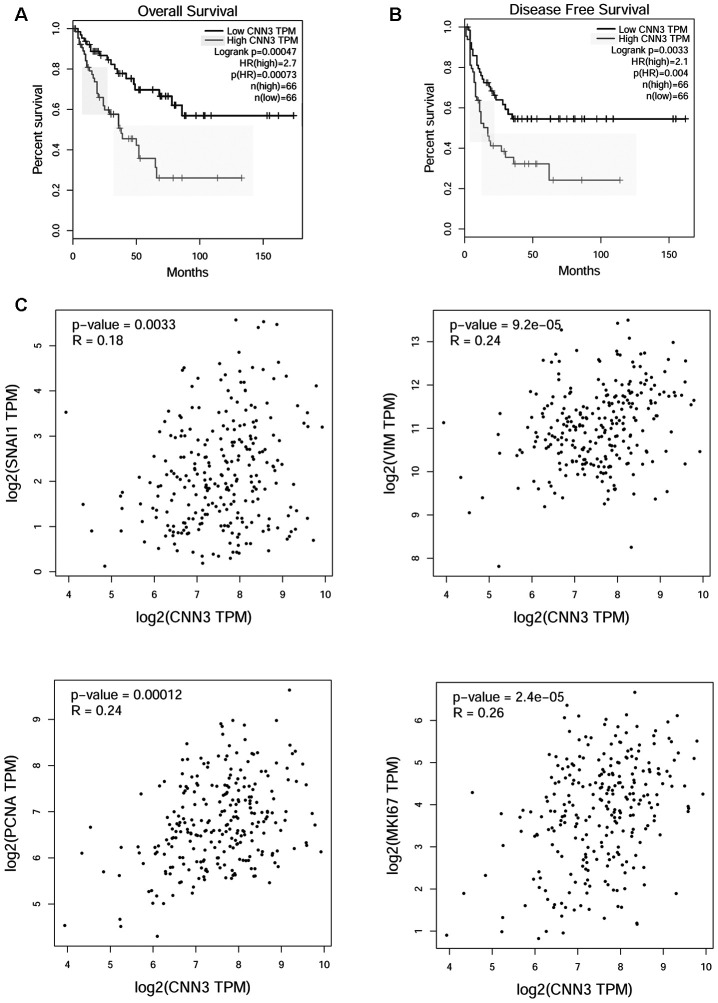
**Functional analysis of CNN3 in TCGA sarcoma cohort analyzed using GEPIA.** (**A**, **B**) Correlation between CNN3 mRNA levels and prognosis. (**C**) Correlation between CNN3 mRNA levels and two mesenchymal markers, Snail and vimentin (VIM), and two tumor proliferation markers, MKI67 and PCNA. TPM: transcripts per million; HR: hazard ratio. The hazard ratio was calculated based on the Cox PH model.

To determine the potential correlation of CNN3 with the clinicopathological features and prognosis, a Pearson χ^2^ test was performed. High CNN3 expression was found to be associated with tumor size (*p*=0.044), tumor stage (*p*=0.002), lymph node metastasis (*p*=0.044), and distant metastasis (p=0.035), but it was not associated with age (*p*=0.089) and sex (*p*=0.291; Table 1). As we only had follow-up information for some enrolled patients, the correlation between CNN3 protein levels and prognosis was analyzed based on the sarcoma gene-expression dataset available on the Gene Expression Profiling Interactive Analysis (GEPIA) database. The results showed that high CNN3 mRNA expression was linked with a poor overall survival (OS; *p*=0.00047) and a shorter disease-free survival (RFS) period (*p*=0.0033; Figure 2A and 2B). The hazard ratio (HR) for OS and RFS was 2.7 (*p*=0.00073) and 2.1 (*p*=0.004), respectively. In addition, CNN3 mRNA levels exhibited a significant positive relationship with the mRNA levels of Snail, vimentin (VIM), MKI67, and PCNA (*p*<0.05; Figure 2C).

**Table 1:** Correlation between CNN3 protein levels and clinicopathological features of patients with osteosarcoma.

**Features**	**n (50)**	**CNN3 protein levels**		**p-value**
		**Low expression (16)**	**High expression (34)**	
Age (years)				0.089
≤25	36	9	27	
> 25	14	7	7	
Sex				0.291
Male	29	11	18	
Female	21	5	16	
Tumor size (cm)				0.044
≤7.5	24	11	13	
>7.5	26	5	21	
Tumor stage				0.002
I+II	22	12	10	
III+IV	28	4	24	
Lymph node metastasis				0.041
No	27	12	15	
Yes	23	4	19	
Distant metastasis				0.035
No	30	13	17	
Yes	20	3	17	

### Effect of CNN3 silencing on cell proliferation *in vitro*

**Figure 3:**
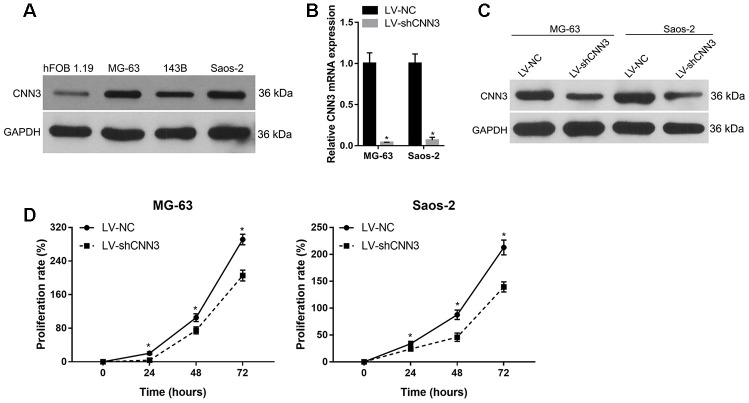
**CNN3 silencing inhibits osteosarcoma cell proliferation.** (**A**) CNN3 expression in osteosarcoma cell lines and the human osteoblast hFOB 1.19 cell line. (**B**, **C**) CNN3 mRNA (**B**) or protein (**C**) levels in LV-shCNN3- or LV-NC-infected MG-63 and Saos-2 cells. (**D**) The proliferation rate (%) of LV-shCNN3- or LV-NC-infected MG-63 and Saos-2 cells at 0, 24, 48, and 72 h. *p<0.05, when LV-shCNN3 vs LV-NC.

CNN3 expression in osteosarcoma cell lines MG-63, 143B, and Saos-2 was higher than that in human osteoblast hFOB 1.19 cells (Figure 3A). Among the three osteosarcoma cell lines, CNN3 expression was higher in MG-63 and Saos-2, therefore these lines were chosen for subsequent assays. qRT-PCR and western blot analyses in CNN3 stable knockdown cells (LV-shCNN3-infected) showed that CNN3 was successfully silenced in MG-63 and Saos-2 (Figure 3B and 3C) cells. The proliferation rate (%) of LV-shCNN3-infected MG-63 and Saos-2 cells at 24, 48, and 72 h was lower than that of LV-NC-infected cells (Figure 3D). These results revealed that CNN3 silencing inhibits the proliferation of osteosarcoma cells *in vitro*.

### Effect of CNN3 silencing on apoptosis and cell cycle *in vitro*

**Figure 4:**
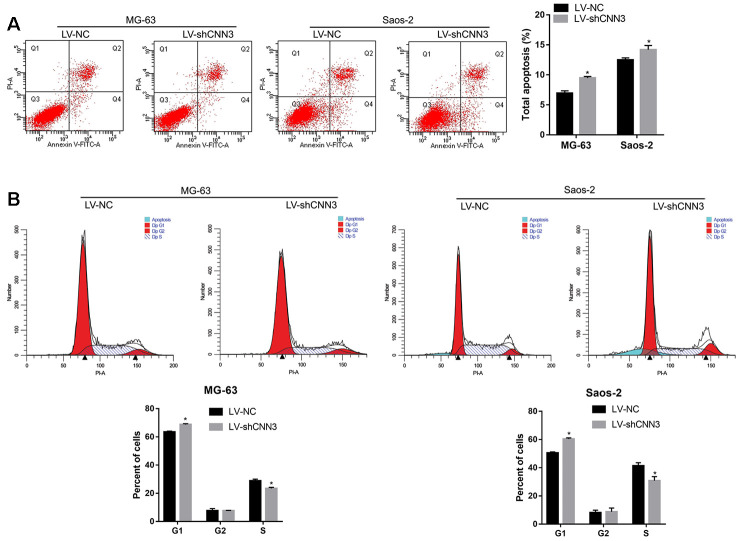
**CNN3 silencing induces apoptosis and G1-stage cell cycle arrest *in vitro*.** (**A**) The effect of CNN3 silencing on apoptosis. Representative graphs of flow cytometric analysis for apoptosis are shown on top. In all four plots, viable cells are seen in the left lower quadrant (FITC^-^/PI^-^); early apoptotic cells in the right lower quadrant (FITC^+^/PI^-^); late apoptotic cells in the right upper quadrant (FITC^+^/PI^+^); and necrotic cells in the left upper quadrant (FITC^-^/PI^+^). Statistical results of the percentage of total apoptotic cells, which are the sum of early and late apoptotic cells are shown below. (**B**) The effect of CNN3 silencing on cell cycle. Graphs showing the flow cytometric analysis for cell cycle stage are on top. The statistical result of cells in G1, G2, and S stages is shown below. *p<0.05, when LV-shCNN3 vs LV-NC.

To evaluate the effect of CNN3 knockdown on apoptosis and cell cycle, a flow cytometric analysis was carried out. As shown in Figure 4A, the number of total apoptotic LV-shCNN3-infected MG-63 and Saos-2 cells was significantly higher than that of LV-NC-infected MG-63 and Saos-2 cells. In addition, the number of LV-shCNN3-infected MG-63 and Saos-2 cells in the G1-stage was significantly higher than that of LV-NC-infected MG-63 and Saos-2 cells, and the number of LV-shCNN3-infected MG-63 and Saos-2 cells in S-stage was significantly lower than that of LV-NC-infected MG-63 and Saos-2 cells (Figure 4B). Although the effects of CNN3 silencing on apoptosis and cell cycle were not pronounced, they were significant. Therefore, these results indicate that CNN3 silencing induces apoptosis and cell cycle arrest in the G1 stage *in vitro*.

### Effect of CNN3 silencing on cell migration and invasion *in vitro*

**Figure 5:**
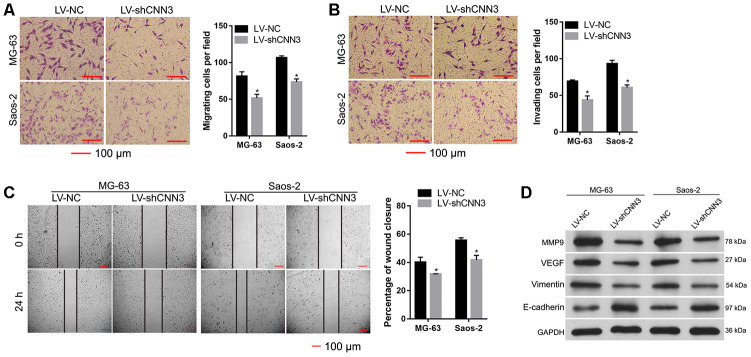
**CNN3 silencing inhibits cell migration and invasion *in vitro*.** (**A**, **B**) Cell migration and invasion were evaluated by a transwell assay (**A**) and the Transwell-Matrigel assay (**B**). Images on the left are representations, and results on the right are the statistical results of cell migration or invasion, where *p<0.05. (**C**) Wound healing assay to evaluate cell migration. Images on the left are representations, and results on the right are the statistical results of wound closure percentage. The wound area was analyzed using the Image Pro-Plus 6.0 software. The percentage of wound closure was calculated using the ratio of wound area at 0 h and 24 h. (**D**) Expression of tumor metastasis-related proteins MMP9, VEGF, vimentin, and E-cadherin was detected by western blotting. *p <0.05, when LV-shCNN3 vs LV-NC.

To evaluate the effect of CNN3 knockdown on cell migration and invasion, transwell migration/invasion assays and wound healing assays were carried out. The results of the transwell assay showed that the number of migrating and invading LV-shCNN3-infected MG-63 and Saos-2 cells was significantly less than that of LV-NC-infected MG-63 and Saos-2 cells (Figure 5A and 5B). Additionally, the wound healing assay demonstrated that wound closure in LV-shCNN3-infected MG-63 and Saos-2 cells was significantly slower than that of LV-NC-infected MG-63 and Saos-2 cells (Figure 5C). Moreover, western blotting was performed to evaluate the effect of CNN3 silencing on the expression of tumor metastasis-related proteins. The results showed that the expression of MMP9, VEGF, and vimentin in LV-shCNN3-infected MG-63 and Saos-2 cells was significantly lower than that in LV-NC-infected MG-63 and Saos-2 cells (Figure 5D), while E-cadherin expression in LV-shCNN3-infected MG-63 and Saos-2 cells was significantly higher than that in LV-NC-infected MG-63 and Saos-2 cells (Figure 5D). MMP9, VEGF, and vimentin are involved in promoting tumor metastasis, while E-cadherin is involved in its suppression [16, 17]. Therefore, these results suggest that CNN3 silencing inhibits cell migration and invasion *in vitro*.

### Effect of CNN3 silencing on ERK1/2 and p38 signaling pathways

**Figure 6:**
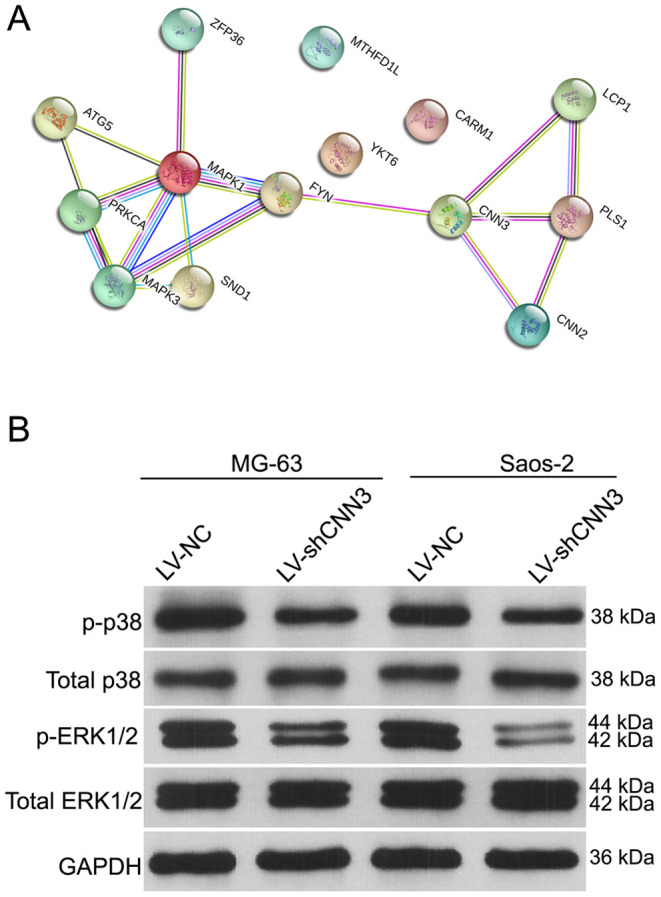
**The effect of CNN3 silencing on the expression of total or phosphorylated p38 and ERK1/2.** (**A**) The STRING interaction network database shows that CNN3 interacts with the MAPK signaling pathways. (**B**) The expression of total or phosphorylated p38 and ERK1/2.

To explore the molecular mechanisms underlying the activity of CNN3, the pathways and interactions of the CNN3 gene were analyzed using the STRING interaction network database (Figure 6A). Western blotting showed that the levels of phosphorylated p38 (p-p38) and phosphorylated extracellular signal-regulated kinase 1 or 2 (p-ERK1/2) were significantly lower in LV-shCNN3-infected MG-63 and Saos-2 cells than those in LV-NC-infected MG-63 and Saos-2 cells (Figure 6B), while the expression of total p38 and p-ERK1/2 was similar between these groups (Figure 6B). These results indicate that CNN3 silencing downregulates the ERK1/2 and p38 signaling pathways.

### Effect of CNN3 silencing on subcutaneous tumor growth and lung metastasis *in vivo*

**Figure 7:**
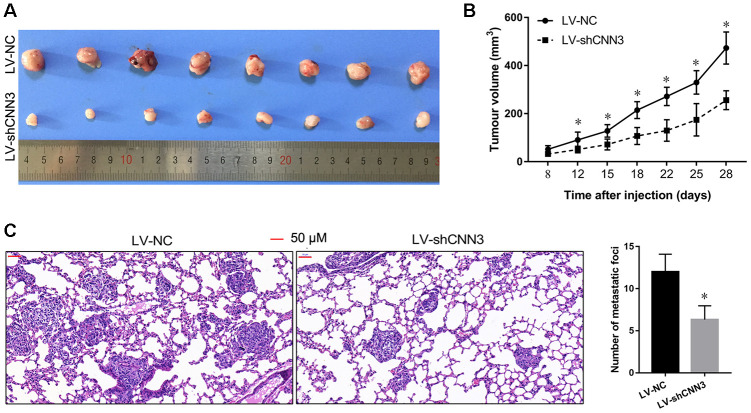
**CNN3 silencing inhibits tumor growth and lung metastasis *in vivo*.** (**A**, **B**) The effect of CNN3 silencing on the growth of subcutaneous xenograft tumors. (**A**) Subcutaneous xenograft tumor graphs. (**B**) Tumor growth curve based on the tumor volume. (**C**) The effect of CNN3 silencing on lung metastases examined by H&E staining. Images on the left are representations. The bar graph showing the statistical evaluation of the number of metastatic foci is on the right. *p<0.05, when LV-shCNN3 vs LV-NC.

To further verify the role of CNN3 *in vivo*, the subcutaneous xenograft tumor mouse model and the lung metastasis mouse model were constructed using the LV-shCNN3-infected and LV-NC-infected MG-63 cells as the Saos-2 cells were not tumorigenic in immunosuppressed mice. The size of the subcutaneous tumor generated by the LV-shCNN3-infected MG-63 cells was smaller than that generated by LV-NC-infected MG-63 cells (Figure 7A). Tumor growth curves also revealed that CNN3 silencing can suppress the growth of subcutaneous tumors (Figure 7B). Furthermore, H&E staining showed that the lung tissues of mice injected with LV-shCNN3-infected MG-63 cells had fewer metastatic foci than those injected with LV-NC-infected MG-63 cells (Figure 7C). These results show that CNN3 silencing inhibits subcutaneous tumor growth and lung metastasis *in vivo*.

## DISCUSSION

In this study, we evaluated the potential diagnostic and prognostic roles of CNN3 in osteosarcoma. We found that CNN3 protein expression was significantly higher in osteosarcoma tissues compared with normal specimens. This was congruent with data showing that CNN3 expression was higher in human osteosarcoma cell lines (MG-63, 143B, and Saos-2) than in the SV40-immortalized normal osteoblastic cell line, hFOB 1.19. In addition, the abnormal expression profile indicated that CNN3 may promote the clinical progression of osteosarcoma, and the function and underlying mechanism of CNN3 in osteosarcoma were further verified.

In the ROC curve analyses, the area under the ROC curve was 0.756. The AUC is an overall measurement of accuracy of biomarkers of diagnostic tests, which help predict disease [18]. An AUC ranging from 0.7 to 0.9 suggests that a given biomarker is highly accurate. Therefore, CNN3 could serve as a potential accurate diagnostic biomarker for patients with osteosarcoma. Furthermore, we found that high CNN3 expression is associated with tumor size, tumor stage, lymph node metastasis, and distant metastasis. Moreover, high CNN3 mRNA levels were significantly linked with poorer OS rates and shorter RFS period. The relative mRNA expression of CNN3 in 14 colorectal cancer tissues was found to be lower than that in normal control tissues, and CNN3 mRNA levels are known to be significantly higher in the Dukes’ stage C group than in the control groups [15]. Moreover, CNN3 has been recognized as one of the more accurate and suitable diagnostic markers for metastatic lymph nodes in colorectal cancer [15]. Taken together, these data support our conclusions in osteosarcoma. However, a limitation in our study is that we did not collect the follow-up information of enrolled patients in this study; instead, the prognostic analysis of CNN3 was based on the information hosted on TCGA. In a future assessment of the prognostic value of CNN3, the levels of the CNN3 protein should be determined.

To investigate the biological function of CNN3 in osteosarcoma progression, the CNN3 gene was knocked down by infection with LV-shCNN3 in osteosarcoma cell lines. Functional *in vitro* data showed that CNN3 silencing inhibited cell proliferation, migration, and invasion. In addition, we found that CNN3 silencing induced apoptosis and caused cell cycle arrest in the G1 stage *in vitro*. Moreover, CNN3 silencing inhibited subcutaneous tumor growth and lung metastases *in vivo*. These results indicate that CNN3 contributes to osteosarcoma progression, which is further supported by previous studies reporting that *CNN3* may function as a potential oncogene in gastric, colon, and cervical cancer [12–14]. Additionally, we found that CNN3 mRNA expression had a significant positive correlation with the mRNA expression of two mesenchymal markers, Snail and vimentin (VIM) [19], and two tumor proliferation markers, MKI67 and PCNA, [20] in sarcoma. These bioinformatic results are consistent with the results of our *in vitro* and in *vivo* experiments, and can further support our conclusion that CNN3 may play an oncogenic role in osteosarcoma progression.

The MAPK signaling pathway is a complex pathway that regulates tumor occurrence and metastasis [21, 22]; it is composed of three main signaling pathways: MAPK/ERK, SAPK/JNK, and p38 MAPK [23]. These can be activated by phosphorylation, and thus are involved in a variety of cellular activities [24, 25]. CNN3 has been shown to increase the phosphorylation of ERK1/2 and p38, but it does not change JNK phosphorylation levels in the human placental trophoblast cell line, BeWo [26]. In the present study, we also found that the phosphorylation of ERK1/2 and p38 is downregulated in CNN3-knockdown cells, suggesting that CNN3 silencing downregulates the ERK1/2 and p38 signaling pathways. Therefore, we hypothesized that CNN3 may be involved in osteosarcoma cell proliferation and metastasis by regulating the MAPK/ERK and p38 MAPK pathways, which play a critical role in osteosarcoma pathogenesis [27]. Previous studies have reported that the suppression of MAPK/ERK and p38 MAPK pathways is an effective approach to inhibit the proliferation and metastasis of osteosarcoma cells [27–29]. However, PI3K/Akt, Wnt, Notch, NF-κB, and p53 are also critical in osteosarcoma pathogenesis [27]. Whether CNN3 can affect these pathways is still unknown, and thus the regulatory mechanism underlying CNN3 activity is far from clear; this is another limitation of our study. In future studies, we will focus on the regulatory mechanisms of CNN3 by measuring mRNA, non-coding RNA, and protein expression in osteosarcoma cell lines.

In conclusion, increased CNN expression is associated with poor prognosis of patients with osteosarcoma. CNN3 may potentially be used as a diagnostic and prognostic biomarker for osteosarcoma and may play an oncogenic role in osteosarcoma progression. We suggest that CNN3 may function through the activation of the ERK1/2 and p38 signaling pathways. In the future, we will attempt to clarify the regulatory mechanisms involved in the potential oncogenic roles of CNN3 in osteosarcoma. Our study provides a potential target for pharmaceutical development and novel therapeutic strategies involving CNN3 inhibition and the control of osteosarcoma growth and metastasis.

## MATERIALS AND METHODS

### Patient information and tissue samples

The medical histories of the fifty osteosarcoma patients enrolled in this study were recorded. This included information about their age, gender, tumor size, tumor stage, node metastasis, and distant metastasis. All patients understood the purpose of our study and gave informed consent. Our study was approved by the ethics committee of the First Affiliated Hospital of Army Medical University (KY201927). Osteosarcoma specimens (n=50) and corresponding normal bone specimens (n=50) from regions around the cancers were obtained at the First Affiliated Hospital of Army Medical University from 2015 to 2017.

### Immunohistochemical staining and analysis

Paraffin-embedded tissues were cut into sections and antigen retrieval was conducted by microwaving a sodium citrate solution (pH 6.0) after dewaxing. After blocking with 5% sheep serum, sections were incubated with rabbit polyclonal CNN3 antibody (1:25; Omnimabs, Alhambra, CA, USA) at 4°C overnight. Next, the 3,3′-diaminobenzidine (DAB)-chromogen, Polymer HRP mouse/rabbit IHC kit, and DAB Chromogenic Substrate Kit were used according to the manufacturer’s instructions (Auragene Biotech, Changsha, China). After staining with a hematoxylin solution, sections were dehydrated, cleared, and mounted with Permount TM Mounting Medium. Immunohistochemical results for CNN3 were judged independently by two senior pathologists, according to the method described previously [30]. The total score for CNN3 staining was generated by multiplying the staining intensity score (0: negative; 1: light yellow; 2: brown; 3: tan) by the percentage of positive cells (0:<5%; 1: 5–25%; 2: 25–50%; 3: 51–75%; 4: >75%). The total score for CNN3 staining results was grouped into two categories, where a score of 1–4 was regarded as low expression, while one of 5–12 was regarded as high expression.

### Bioinformatic analysis

The CNN3 mRNA expression levels in the sarcoma cohort were analyzed using GEPIA (http://gepia2.cancer-pku.cn/#index), a web-based tool that delivers fast and customizable functionalities based on TCGA and GTEx data [31]. The correlation between CNN3 mRNA levels and prognosis, and between CNN3 mRNA expression and mRNA expression levels of Snail, vimentin (VIM), MKI67, and PCNA; were analyzed using the GEPIA database [31]. The pathways and interactions for the CNN3 gene were analyzed using the STRING interaction network database (https://version11.string-db.org/cgi/network.pl?taskId=02KGNUORuL3B).

### Cell culture

SV40-immortalized normal osteoblastic cell line (hFOB 1.19) and human osteosarcoma cell lines (MG-63, 143B, and Saos-2) were purchased from ATCC, the Global Bioresource Center (Manassas, VA, USA), and cultured according to the protocol of the supplier.

### Construction of CNN3 stable knockdown cells

The shRNA-mediated lentivirus targeting a sequence of CNN3 (CCCTACAGATGGGTACCAA; LV-shCNN3) was purchased from Hanbio Biotechnology Co. Ltd. (Shanghai, China). Lentiviruses that were packaged with the empty vector served as the negative control (LV-NC). MG-63 and Saos-2 cells were infected with LV-shCNN3 or LV-NC, with a multiplicity of infection of 100 and selection by puromycin.

### Quantitative reverse transcription-polymerase chain reaction (qRT-PCR)

MG-63 and Saos-2 cells infected with LV-shCNN3 or LV-NC were seeded (4×10^5^ cells/well) in 6-well plates. After being cultured for 48 h, cells were harvested for total RNA isolation using Trizol (Invitrogen, Carlsbad, CA, USA). Next, cDNA synthesis was performed using the M-MLV Reverse Transcriptase Kit (Promega, Madison, WI, USA), and qPCR was performed using the SYBR Green qPCR SuperMix (Invitrogen) on the ABI PRISM® 7500 Sequence Detection System (Applied Biosystems, Foster City, CA, USA). The relative expression level of CNN3 was calculated using the 2^−ΔΔCT^ method [32]. The internal control was 18S rRNA. The primers for CNN3 and 18S are as follows: CNN3-F: CCCAGAAAGGAATGAGTGTGT, CNN3-R: CTCGCCATGATACTCATCAG, 18S-F: CCTGGATACCGCAGCTAGGA, and 18S-R: GCGGCGCAATACGAATGCCCC.

### Western blot

MG-63 and Saos-2 cells infected with LV-shCNN3 or LV-NC were seeded (4 × 10^5^ cells/well) in 6-well plates. After being cultured for 48 h, cells were harvested, and the total protein content was isolated with RIPA buffer. After protein quantification, 30 μg of total protein was used for western blotting, which was conducted according to the methods described previously [33]. The primary antibodies used in this study were purchased from Abcam (Cambridge, MA, USA) and their details are as follows: anti-CNN3 (1:2000, ab151427), anti-MMP9 (1:1500, ab76003), anti-VEGF (1:5000, ab52917), anti-vimentin (1:1000, ab92547), anti-E-cadherin (1:1000, ab231303), p-p38 (1:1000, ab45381), p38 (1:1000, ab32142), ERK1/2 (1:1000, ab115799), p-ERK1/2 (1:800, ab214362), and anti-GAPDH (1:5000, ab181602).

### Proliferation assays and apoptosis assays

Proliferation assays were carried out using the Cell Counting Kit-8 (CCK-8; Beyotime Institute of Biotechnology, Shanghai, China) as described previously [33]. Briefly, MG-63 and Saos-2 cells infected with LV-shCNN3 or LV-NC were seeded (4 × 10^4^ cells/well) in 96-well plates. The optical density (OD) value was detected at 0, 24, 48, and 72 h after seeding. The proliferation rate (%) was calculated using the formula: proliferation rate (%) = *OD value*_time point_ *÷OD value*_0 h_ *× 100% (same group).* After seeding into 6-well plates and incubating for 48 h, cells were harvested and apoptosis assays were carried out using the Annexin V-FITC apoptosis detection kit (BD Biosciences, Franklin Lake, NJ, USA), as described previously [33]. The number of apoptotic cells was analyzed using the BD FACSDiva 8.0.1 software.

### Cell cycle assays

MG-63 and Saos-2 cells infected with LV-shCNN3 or LV-NC were seeded (4 × 10^5^ cells/well) in 6-well plates. After being cultured for 48 h, cells were harvested, and the cell cycle distribution was analyzed using the Cell Cycle Analysis Kit (Beyotime, Shanghai, China) as described previously [34]. Cell cycle stages were analyzed using the BD LSRII Flow Cytometer (BD LSRII, San Jose, CA, USA).

### Transwell assays

MG-63 and Saos-2 cells infected with LV-shCNN3 or LV-NC were seeded (1 × 10^5^ cells in 200 μL of serum-free medium) in the upper chamber of an insert (pore size, 8 μm; Becton Dickinson Labware). For invasion assay, the upper chambers were pre-coated with Matrigel (BD Biosciences). The lower chamber was filled with 600 μL of 20% fetal bovine serum medium. After 24 h incubation, cells in the upper chamber or Matrigel were removed with a cotton swab, and cells on the underside were fixed with 4% paraformaldehyde at 4°C for 10 min, cells were then stained with 0.1% crystal violet in 20% ethanol for 20 min. The number of migrated or invasive cells were counted in ten randomly selected fields using a phase-contrast microscope. The assays were performed in triplicate.

### Wound healing assay

MG-63 and Saos-2 cells infected with LV-shCNN3 or LV-NC were seeded (5×10^5^ cells/well) into 6-well plates with straight lines at the bottom of the plate. The next day, a wound was generated along the five straight lines using a sterile 200 μL pipette tip. After washing with phosphate-buffered saline (PBS) three times, cells were cultured in serum-free medium at 37°C in humidified air with 5% CO_2_. Cell migration into the wound area was monitored and photographed at 24 h. The wound area was analyzed by using the Image Pro-Plus 6.0 software (Media Cybernetics, Rockville, MD, USA). The percentage of wound closure was calculated using the formula: 1-wound area _24 h_ *÷* wound area _0 h_ *× 100%*.

### Subcutaneous xenograft and pulmonary metastasis model in nude mice

Six-week-old athymic nude mice (n=32) were purchased from Beijing Vital River Laboratory Animal Technology Co. Ltd. (Beijing, China). All mice were housed in a specific pathogen-free animal house and provided free access to food and water. All animal experiments in this study were approved by the ethics committee of the First Affiliated Hospital of Army Medical University (AMUWEC202023).

To construct subcutaneous xenograft models, 16 nude mice were divided into two groups (n=8), subcutaneously injected in the right armpit region with MG-63 cells infected with LV-shCNN3 or LV-NC (4×10^6^ cells in 0.2 mL of PBS). After injection for 8, 15, 18, 22, 26, and 28 days, the maximum transcutaneous diameter (tumor length, L) and the corresponding maximum vertical transcutaneous diameter (tumor width, W) of the subcutaneous tumor were measured using vernier calipers, and the tumor volume was calculated using the formula: ½ × L × W^2^ [35].

To construct pulmonary metastasis models, 16 nude mice were divided into two groups (n=8), intravenous tail injected with 5 × 10^5^ MG-63 cells infected with LV-shCNN3 or LV-NC, which were resuspended in 100 μL Hank’s Balanced Salt Solution. Six weeks later, all mice were euthanized by intraperitoneal injection with sodium pentobarbital (130 mg/kg). Lung tissues were isolated and fixed in 4% paraformaldehyde. Hematoxylin and eosin (H&E) staining was performed to evaluate the status of lung metastasis. Six randomly selected fields were photographed for each section, and the number of metastatic foci in each photograph were counted. The average number of metastatic foci was used as the number of metastatic foci of each group in lung tissues.

### Statistical analysis

Statistical analyses were performed using GraphPad Prism 7.0 version (GraphPad Software, Inc., San Diego, CA, USA). The results are shown as mean ± standard deviation. Non-parametric tests were used to compare the total score for CNN3 staining in osteosarcoma specimens and normal specimens. Statistical comparisons between the two groups of functional experiments *in vitro* and *in vivo* were analyzed using unpaired *t*-tests. The significance of the correlation of CNN3 expression with clinicopathological features was determined by a Pearson χ^2^ test. The receiver operating characteristic (ROC) curve and the area under the ROC curve analyses were performed to assess the diagnostic value of CNN3 using GraphPad Prism 7.0 version, and the optimal CNN3 cut-off value for diagnosis was obtained based on the Youden index according to the ROC curves. A *p*< 0.05 was considered statistically significant.

## References

[1] Biazzo A, De Paolis M. Multidisciplinary approach to osteosarcoma. Acta Orthop Belg. 2016; 82:690–98. 29182106

[2] Guillon MA, Mary PM, Brugière L, Marec-Bérard P, Pacquement HD, Schmitt C, Guinebretière JM, Tabone MD. Clinical characteristics and prognosis of osteosarcoma in young children: a retrospective series of 15 cases. BMC Cancer. 2011; 11:407. 10.1186/1471-2407-11-407 21942935 PMC3188515

[3] Picci P. Osteosarcoma (osteogenic sarcoma). Orphanet J Rare Dis. 2007; 2:6. 10.1186/1750-1172-2-6 17244349 PMC1794406

[4] Siegel RL, Miller KD, Jemal A. Cancer statistics, 2018. CA Cancer J Clin. 2018; 68:7–30. 10.3322/caac.21442 29313949

[5] Mialou V, Philip T, Kalifa C, Perol D, Gentet JC, Marec-Berard P, Pacquement H, Chastagner P, Defaschelles AS, Hartmann O. Metastatic osteosarcoma at diagnosis: prognostic factors and long-term outcome—the french pediatric experience. Cancer. 2005; 104:1100–09. 10.1002/cncr.21263 16015627

[6] Liu R, Jin JP. Calponin isoforms CNN1, CNN2 and CNN3: regulators for actin cytoskeleton functions in smooth muscle and non-muscle cells. Gene. 2016; 585:143–53. 10.1016/j.gene.2016.02.040 26970176 PMC5325697

[7] Shibukawa Y, Yamazaki N, Kumasawa K, Daimon E, Tajiri M, Okada Y, Ikawa M, Wada Y. Calponin 3 regulates actin cytoskeleton rearrangement in trophoblastic cell fusion. Mol Biol Cell. 2010; 21:3973–84. 10.1091/mbc.E10-03-0261 20861310 PMC2982094

[8] Daimon E, Shibukawa Y, Wada Y. Calponin 3 regulates stress fiber formation in dermal fibroblasts during wound healing. Arch Dermatol Res. 2013; 305:571–84. 10.1007/s00403-013-1343-8 23545751

[9] Yamane T, Asanoma K, Kobayashi H, Liu G, Yagi H, Ohgami T, Ichinoe A, Sonoda K, Wake N, Kato K. Identification of the critical site of calponin 1 for suppression of ovarian cancer properties. Anticancer Res. 2015; 35:5993–99. 26504022

[10] Hu J, Xie W, Shang L, Yang X, Li Q, Xu M, Dou J, Zhou Y, Niu W, Wu Y. Knockdown of calponin 2 suppressed cell growth in gastric cancer cells. Tumour Biol. 2017; 39:1010428317706455. 10.1177/1010428317706455 28714360

[11] Guo QC, Shen JN, Jin S, Wang J, Huang G, Zhang LJ, Huang G, Yin JQ, Zou CY, Li MT. Comparative proteomic analysis of human osteosarcoma and SV40-immortalized normal osteoblastic cell lines. Acta Pharmacol Sin. 2007; 28:850–58. 10.1111/j.1745-7254.2007.00603.x 17506944

[12] Hong KS, Kim H, Kim SH, Kim M, Yoo J. Calponin 3 regulates cell invasion and doxorubicin resistance in gastric cancer. Gastroenterol Res Pract. 2019; 2019:3024970. 10.1155/2019/3024970 30911294 PMC6398029

[13] Nair VA, Al-Khayyal NA, Sivaperumal S, Abdel-Rahman WM. Calponin 3 promotes invasion and drug resistance of colon cancer cells. World J Gastrointest Oncol. 2019; 11:971–82. 10.4251/wjgo.v11.i11.971 31798778 PMC6883188

[14] Xia L, Yue Y, Li M, Zhang YN, Zhao L, Lu W, Wang X, Xie X. CNN3 acts as a potential oncogene in cervical cancer by affecting RPLP1 mRNA expression. Sci Rep. 2020; 10:2427. 10.1038/s41598-020-58947-y 32051425 PMC7016181

[15] Nakarai C, Osawa K, Akiyama M, Matsubara N, Ikeuchi H, Yamano T, Hirota S, Tomita N, Usami M, Kido Y. Expression of AKR1C3 and CNN3 as markers for detection of lymph node metastases in colorectal cancer. Clin Exp Med. 2015; 15:333–41. 10.1007/s10238-014-0298-1 24934327 PMC4522272

[16] Yeung KT, Yang J. Epithelial-mesenchymal transition in tumor metastasis. Mol Oncol. 2017; 11:28–39. 10.1002/1878-0261.12017 28085222 PMC5242415

[17] Ribatti D. Tumor refractoriness to anti-VEGF therapy. Oncotarget. 2016; 7:46668–77. 10.18632/oncotarget.8694 27081695 PMC5216828

[18] Yin J, Tian L. Joint confidence region estimation for area under ROC curve and youden index. Stat Med. 2014; 33:985–1000. 10.1002/sim.5992 24123069

[19] Hao Y, Baker D, Ten Dijke P. TGF-β-mediated epithelial-mesenchymal transition and cancer metastasis. Int J Mol Sci. 2019; 20:2767. 10.3390/ijms20112767 31195692 PMC6600375

[20] Wagner M, Girod SC, Guenther A, Krueger G. (1996). MDM2 Ki67, PCNA, p53, and viruses in oropharyngeal tumors.

[21] Pan H, Wang Y, Na K, Wang Y, Wang L, Li Z, Guo C, Guo D, Wang X. Autophagic flux disruption contributes to Ganoderma lucidum polysaccharide-induced apoptosis in human colorectal cancer cells via MAPK/ERK activation. Cell Death Dis. 2019; 10:456. 10.1038/s41419-019-1653-7 31186406 PMC6560101

[22] Ham J, Lim W, Kim K, Heo YM, Ryu SM, Lee D, Kim JJ, Song G. Gentisyl Alcohol Inhibits Proliferation and Induces Apoptosis via Mitochondrial Dysfunction and Regulation of MAPK and PI3K/AKT Pathways in Epithelial Ovarian Cancer Cells. Mar Drugs. 2019; 17:331. 10.3390/md17060331 31163640 PMC6627157

[23] Klein AM, Zaganjor E, Cobb MH. Chromatin-tethered MAPKs. Curr Opin Cell Biol. 2013; 25:272–77. 10.1016/j.ceb.2013.01.002 23434067 PMC3654648

[24] Séverin S, Ghevaert C, Mazharian A. The mitogen-activated protein kinase signaling pathways: role in megakaryocyte differentiation. J Thromb Haemost. 2010; 8:17–26. 10.1111/j.1538-7836.2009.03658.x 19874462

[25] Keshet Y, Seger R. The MAP kinase signaling cascades: a system of hundreds of components regulates a diverse array of physiological functions. Methods Mol Biol. 2010; 661:3–38. 10.1007/978-1-60761-795-2_1 20811974

[26] Appel S, Ankerne J, Appel J, Oberthuer A, Mallmann P, Dötsch J. CNN3 regulates trophoblast invasion and is upregulated by hypoxia in BeWo cells. PLoS One. 2014; 9:e103216. 10.1371/journal.pone.0103216 25050546 PMC4106885

[27] Kushlinskii NE, Fridman MV, Braga EA. Molecular mechanisms and microRNAs in osteosarcoma pathogenesis. Biochemistry (Mosc). 2016; 81:315–28. 10.1134/S0006297916040027 27293089

[28] Wang C, Zhou X, Li W, Li M, Tu T, Ba X, Wu Y, Huang Z, Fan G, Zhou G, Wu S, Zhao J, Zhang J, et al. Macrophage migration inhibitory factor promotes osteosarcoma growth and lung metastasis through activating the RAS/MAPK pathway. Cancer Lett. 2017; 403:271–279. 10.1016/j.canlet.2017.06.011 28642171

[29] Yu Y, Li Y, Zhou L, Yang G, Wang M, Hong Y. Cryptochrome 2 (CRY2) Suppresses Proliferation and Migration and Regulates Clock Gene Network in Osteosarcoma Cells. Med Sci Monit. 2018; 24:3856–3862. 10.12659/MSM.908596 29879092 PMC6020744

[30] Zhang H, Pan YZ, Cheung M, Cao M, Yu C, Chen L, Zhan L, He ZW, Sun CY. LAMB3 mediates apoptotic, proliferative, invasive, and metastatic behaviors in pancreatic cancer by regulating the PI3K/Akt signaling pathway. Cell Death Dis. 2019; 10:230. 10.1038/s41419-019-1320-z 30850586 PMC6408539

[31] Tang Z, Li C, Kang B, Gao G, Li C, Zhang Z. GEPIA: a web server for cancer and normal gene expression profiling and interactive analyses. Nucleic Acids Res. 2017; 45:W98–102. 10.1093/nar/gkx247 28407145 PMC5570223

[32] Livak KJ, Schmittgen TD. Analysis of relative gene expression data using real-time quantitative PCR and the 2(-delta delta C(T)) method. Methods. 2001; 25:402–08. 10.1006/meth.2001.1262 11846609

[33] Song L, Zhou Z, Gan Y, Li P, Xu Y, Zhang Z, Luo F, Xu J, Zhou Q, Dai F. Long noncoding RNA OIP5-AS1 causes cisplatin resistance in osteosarcoma through inducing the LPAATβ/PI3K/AKT/mTOR signaling pathway by sponging the miR-340-5p. J Cell Biochem. 2019; 120:9656–66. 10.1002/jcb.28244 30548308

[34] Li J, Fang R, Gong Q, Wang J. miR-99b suppresses IGF-1R expression and contributes to inhibition of cell proliferation in human epidermal keratinocytes. Biomed Pharmacother. 2015; 75:159–64. 10.1016/j.biopha.2015.07.013 26297545

[35] Euhus DM, Hudd C, LaRegina MC, Johnson FE. Tumor measurement in the nude mouse. J Surg Oncol. 1986; 31:229–34. 10.1002/jso.2930310402 3724177

